# Improved Energy Storage Density and Efficiency of Nd and Mn Co-Doped Ba_0.7_Sr_0.3_TiO_3_ Ceramic Capacitors Via Defect Dipole Engineering

**DOI:** 10.3390/ma16206753

**Published:** 2023-10-18

**Authors:** Hyunsu Choi, Srinivas Pattipaka, Yong Hoon Son, Young Min Bae, Jung Hwan Park, Chang Kyu Jeong, Han Eol Lee, Sung-Dae Kim, Jungho Ryu, Geon-Tae Hwang

**Affiliations:** 1 Department of Materials Science and Engineering, Pukyong National University, 45, Yongso-Ro, Nam-Gu, Busan 48513, Republic of Korea; sky5021184@pukyong.ac.kr (H.C.); cnuphy444@gmail.com (S.P.); mike4009@pukyong.ac.kr (Y.H.S.); dud1560@pukyong.ac.kr (Y.M.B.); sdkim@pknu.ac.kr (S.-D.K.); 2 Department of Mechanical Engineering, Department of Aeronautics, Mechanical and Electronic Convergence Engineering, Kumoh National Institute of Technology, 61 Daehak-Ro, Gumi 39177, Republic of Korea; parkjh1151@kumoh.ac.kr; 3 Division of Advanced Materials Engineering, Jeonbuk National University, Jeonju 54896, Republic of Korea; ckyu@jbnu.ac.kr (C.K.J.); haneol@jbnu.ac.kr (H.E.L.); 4 School of Materials Science and Engineering, Yeungnam University, 280 Daehak-Ro, Gyeongsan-si 38541, Republic of Korea; jhryu@ynu.ac.kr

**Keywords:** ceramic capacitors, donor–acceptor complex, defect dipole engineering, dielectric and ferroelectric properties, energy storage density and efficiency

## Abstract

In this paper, we investigate the structural, microstructural, dielectric, and energy storage properties of Nd and Mn co-doped Ba_0.7_Sr_0.3_TiO_3_ [(Ba_0.7_Sr_0.3_)_1−*x*_Nd*_x_*Ti_1−*y*_Mn*_y_*O_3_ (BSNTM) ceramics (*x* = 0, 0.005, and *y* = 0, 0.0025, 0.005, and 0.01)] via a defect dipole engineering method. The complex defect dipoles ${(Mn}_{Ti}^{”}-V_{O}^{∙∙}{)}^{∙}$ and ${(Mn}_{Ti}^{”}-V_{O}^{∙∙})$ between acceptor ions and oxygen vacancies capture electrons, enhancing the breakdown electric field and energy storage performances. XRD, Raman, spectroscopy, XPS, and microscopic investigations of BSNTM ceramics revealed the formation of a tetragonal phase, oxygen vacancies, and a reduction in grain size with Mn dopant. The BSNTM ceramics with *x* = 0.005 and *y* = 0 exhibit a relative dielectric constant of 2058 and a loss tangent of 0.026 at 1 kHz. These values gradually decreased to 1876 and 0.019 for *x* = 0.005 and *y* = 0.01 due to the Mn^2+^ ions at the Ti^4+^- site, which facilitates the formation of oxygen vacancies, and prevents a decrease in Ti^4+^. In addition, the defect dipoles act as a driving force for depolarization to tailor the domain formation energy and domain wall energy, which provides a high difference between the maximum polarization of *P_max_* and remnant polarization of *P_r_* (Δ*P* = 10.39 µC/cm^2^). Moreover, the complex defect dipoles with optimum oxygen vacancies in BSNTM ceramics can provide not only a high Δ*P* but also reduce grain size, which together improve the breakdown strength from 60.4 to 110.6 kV/cm, giving rise to a high energy storage density of 0.41 J/cm^3^ and high efficiency of 84.6% for *x* = 0.005 and *y* = 0.01. These findings demonstrate that defect dipole engineering is an effective method to enhance the energy storage performance of dielectrics for capacitor applications.

## 1. Introduction

Dielectric capacitors are key components of pulsed power applications, and are extensively used in microwave communications, electromagnetic devices, hybrid electric vehicles, and high-frequency inverters [1,2,3,4,5]. Notably, dielectric capacitors display ultrahigh power density, ultrafast charge–discharge rates, excellent fatigue resistance, and thermal stability as compared to batteries [6,7,8]. However, their energy storage density performance is lower than that of batteries because of their low breakdown strength (BDS), which limits their applications in energy storage devices [9,10,11]. It is thus necessary to develop new dielectric capacitors with high energy storage density and high energy efficiency to meet the increasing demands for energy storage devices.

**Figure 1:**
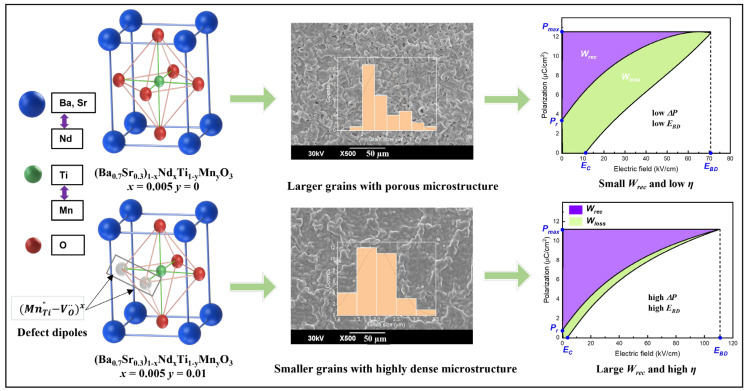
Schematic illustration for energy storage performance of Nd and Mn co-doped BST ceramics. Defect dipoles ${(Mn}_{Ti}^{”}-V_{O}^{∙∙}{)}^{x}$ between acceptor ions and oxygen vacancies capture electrons, reduce grain size, and provide a high difference between *P_max_* and *P_r_*, which improve the breakdown electric field with Mn, resulting in a high energy storage density and high energy efficiency in BSNTM ceramics.

The key parameters for energy storage in dielectric capacitors, such as the total energy storage density (*W_tot_*), recoverable energy density (*W_rec_*), and energy efficiency (*η*) can be calculated by the following equations [8,9,12]:

(1) $$W_{tot}=\int_{0}^{P_{max}}E\;dP$$

(2) $$W_{rec}=\int_{P_{r}}^{P_{max}}E\;dP$$

(3) $$\eta =\frac{W_{rec}}{W_{rec+W_{loss}}}\times 100\%$$

where *E* is the applied electric field, *P* is induced polarization, *P_max_* is maximum polarization, *P_r_* is remnant polarization, and *W_loss_* is hysteresis loss (Figure 1). According to these equations, *W_rec_* and *η* can be improved by increasing the difference between *P_max_* and *P_r_* (Δ*P = P_max_ − P_r_*) and the BDS/breakdown electric field (*E_BD_*), which means that energy storage mostly depends on the Δ*P* and *E_BD_* parameters, hence a larger *E_BD_* is the cause of high energy storage density. Normally, high-dielectric-constant materials with a large *P_max_* display high dielectric loss, which leads to low BDS and *W_rec_* [13]. Researchers have sought to enhance BDS by modifying extrinsic properties, such as reducing the thickness of dielectric capacitors [14,15], porosity [16,17], and grain size [18,19], and adopting a core–shell structure [20,21]. They have also modified intrinsic properties, including enhancing the bandgap energy [9,22], tailoring electrical homogeneity, and reducing electrical conductivity [23].

In recent years, lead-free dielectric capacitors have received significant attention, and a great deal of research has been carried out to enhance energy storage properties due to lead toxicity and environmental issues. Lead-free dielectrics, such as BaTiO_3_ (BT) [15,24,25], Bi_0.5_Na_0.5_TiO_3_ (BNT) [13,26,27,28,29], BiFeO_3_ (BFO) [1,23,30], and K_0.5_Na_0.5_NbO_3_ (KNN) [31,32]-based materials/composites, afford improved energy storage performance and energy efficiency for energy storage applications. In particular, BT-based ceramics are potential candidates and are widely used for capacitor applications due to their high polarization, high dielectric constant, and low Curie temperature (*T_C_*) [33,34]. Few oxide materials (Al_2_O_3_, SiO_2_, and MgO) are used as additives to improve the BDS and energy storage properties of BT-based ceramics [35,36,37]. Kovbasiuk et al. [38] investigated the thermophysical properties of PbO–ZnO–B_2_O_3_ with the doping of Al_2_O_3_, SiO_2,_ and BaO oxides for dielectric layers on film-heating elements. Rafik et al. [39] reported Sr substitution at the A-site of BT (Ba_0.7_Sr_0.3_TiO_3_) ceramics and improved dielectric properties. However, oxygen vacancies and conduction electrons can occur during the sintering process of BT-based ceramics at high temperatures, resulting in a high dielectric loss [40].

Aliovalent doping is an effective method for tailoring the electrical properties of oxide materials. The use of donor dopants, such as La and Nd, is an effective approach to compensate for the formation of oxygen vacancies to improve the dielectric properties of BT ceramics. Morison and Shaikh et al. [41,42] reported La- and Nd-doped BT ceramics with a high dielectric constant of 25,000 and 13,000 at *T_C_*, respectively. On the other hand, acceptor (Mn^2+^ at Ti^4+^-site) doping in BT ceramics promotes the formation of oxygen vacancies and minimizes the decrease in Ti^4+^ during the sintering process in low-oxygen atmospheres. Therefore, Mn-doped BT decreases dielectric loss [43,44,45]. Recently, Yueshun et al. [46] demonstrated defect dipoles via oxygen vacancies in acceptor-doped (specifically Fe) Sr_2_Bi_4_Ti_(5 − *x*)_Fe*_x_*O_18_ (*x* = 0.04–0.12), and enhanced *E_BD_* and energy storage properties.

In this paper, we present a defect dipole engineering method to improve the breakdown strength and energy storage performance by co-doping Nd and Mn in Ba_0.7_Sr_0.3_TiO_3_ (BST) ceramics that have been prepared via the traditional solid-state reaction method. Nd-doped BST [(Ba_0.7_Sr_0.3_)_1 − *x*_Nd*_x_*TiO_3_, BSNT] ceramics can compensate for the formation of oxygen vacancies, improving the dielectric constant of BSNT ceramics. In contrast, Mn-doped BSNT ceramics [(Ba_0.7_Sr_0.3_)_1 − *x*_Nd*_x_*Ti_1 − *y*_Mn*_y_*O_3_, BSNTM] facilitate the formation of oxygen vacancies, prevent a decrease in Ti^4+^, and yield low dielectric loss. Therefore, simultaneously, a high dielectric constant and low dielectric loss can be expected with Nd and Mn co-dopants in BST. Moreover, complex defect dipoles with uniform and small-grained microstructure provide a high difference between *P_max_* and *P_r_* (Δ*P*~10.39 µC/cm^2^); these show the improved breakdown strength of 110.6 kV/cm with Nd and Mn, which results in a high energy storage density of 0.41 J/cm^3^ and high efficiency of 84.6% in BSNTM ceramics, as schematically shown in Figure 1.

## 2. Materials and Methods

(Ba_0.7_Sr_0.3_)_1 − *x*_Nd*_x_*Ti_1 − *y*_Mn*_y_*O_3_ (BSNTM) (*x* = 0, 0.005, and *y* = 0, 0.0025, 0.005, and 0.01) lead-free ceramics were synthesized using the traditional solid-state reaction method. The raw materials BaCO_3_ (Sigma-Aldrich, St. Louis, MO, USA, 99%), SrCO_3_ (Sigma-Aldrich, 98%), Nd_2_O_3_ (Sigma-Aldrich, 99.9%), TiO_2_, (Sigma-Aldrich, 99%), and MnO_2_ (Sigma-Aldrich, 99%) were weighed in stoichiometric proportions and ball-milled for 24 h. After drying the slurry, the BSNTM powder was calcined at 1150 ^o^C for 3 h to obtain the phase of BSNTM. Further, 3 wt.% of Li_2_CO_3_ (Junsei, London, UK, 99%) powder was added to this calcined powder as a sintering aid, and the powder was again ball-milled for 12 h to reduce the sintering temperature and increase its bulk density. Subsequently, 5 wt.% of polyvinyl alcohol (Sigma-Aldrich, 99%) was added, and the powder was pressed into pellets with dimensions of 10 mm in diameter and 0.5 mm in thickness at a pressure of 10 MPa, followed by sintering at 1050 °C for 2 h. Finally, silver paste (ELCOAT, Electroconductives) was applied on both surfaces of the prepared pellets of BSNTM to carry out electrical characterizations.

The crystal structure of the BSNTM samples was tested using an X-ray diffractometer (Rigaku, Tokyo, Japan, Ultima IV) with Cu-Kα radiation (λ = 1.5406 Å) and a Raman spectrometer (JASCO, Tokyo, Japan, NRS-5100) with 532 nm excitation. A scanning electron microscope (SEM, TESCAN, Brno—Kohoutovice, Czech Republic, VEGA II LSU) equipped with an energy-dispersive spectrometer (EDS), and SE (secondary electron) ET (Everhart–Thornley)-type detector (YAG crystal) was used to examine the surface morphology, composition mapping, and elemental distribution at an accelerating voltage of 30 kV. Room-temperature (RT) dielectric properties were measured in the frequency range of 100 Hz–100 kHz using an impedance analyzer (Hewlett Packard, Palo Alto, CA, USA, 4294A). Ferroelectric properties (*P-E* loops) were measured using a ferroelectric tester (Aix ACT, TF Analyzer 2000). The chemical states of the BSNTM samples were measured using X-ray photoelectron spectroscopy (XPS; KRATOS Analytical Ltd., Manchester, UK, AXIS SUPRA).

## 3. Results and Discussion

### 3.1. Phase Formation and Crystal Structure

**Figure 2:**
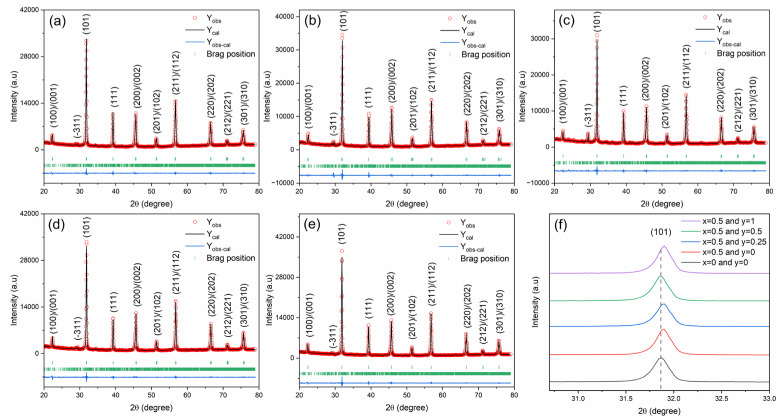
Rietveld refined XRD patterns of (Ba_0.7_Sr_0.3_)_1 − *x*_Nd*_x_*Ti_1 − *y*_Mn*_y_*O_3_ ceramics for (**a**) *x* = 0 and *y* = 0, (**b**) *x* = 0.005 and *y* = 0, (**c**) *x* = 0.005 and *y* = 0.0025, (**d**) *x* = 0.005 and *y* = 0.005, and (**e**) *x* = 0.005 and *y* = 0.01. Figure (**f**) shows a shift in the (101) diffraction peak in the 2θ range from 30.7 − 33°.

Figure 2a–e show the Rietveld refinement X-ray diffraction (XRD) patterns of BSNTM ceramics for *x* = 0 and 0.005, and *y* = 0, 0.0025, 0.005, and 0.01, in the 2θ range of 20–80°. The Rietveld refinement XRD analysis was carried out to confirm the phase and peak profiles by fitting the pseudo-Voigt function using full-proof software. All the samples exhibited a tetragonal BSNTM phase (P4 mm) with a small secondary phase of triclinic Li_2_O (P1). The phase fraction of the BSNTM phase initially decreased from 94.41 to 87.73% for *x* = 0.005 and *y* = 0.0025, and further increased to 95.16% with the Mn concentration (Table 1). At RT, Ba_1 − *x*_Sr*_x_*TiO_3_ ceramics exhibit a tetragonal crystal structure for *x* = 0.3, as reported by Rafik et al. [39]. From the XRD results, there is no peak splitting/merging observed with the substitution of Nd and Mn into BST ceramics due to the lack of changes in the tetragonal crystal structure and the very low doping concentration of Nd and Mn (*x* = 0.005 and *y* = 0.0025–0.01). In Figure 2f, it can be seen that the position of the predominant (101) diffraction peak shifted towards higher angles with Nd for *x* = 0.005 and *y* = 0, and it shifted back to lower angles with Mn into BST for *x* = 0.005 and *y* = 0.0025–0.01. The shift towards lower and higher angles in the diffraction peak demonstrates an increase and decrease in the lattice cell parameters due to the incorporation of Nd and Mn in the BST system, respectively. The determined lattice cell parameters and lattice volume of all the samples are listed in Table 1. For *x* = 0.005 and *y* = 0, the Nd^3+^ (1.27 Å) ions can be occupied at the A-site of Ba^2+^ (1.61 Å) and Sr^2+^ (1.12 Å), whereas Mn^2+^ (0.66 Å) ions can occupied at the B-site of the Ti^4+^ (0.60 Å) site of the BST system for *x* = 0.005 and *y* = 0.0025–0.01, due to their mismatch of ionic radii and valences [39,47].

**Figure 3:**
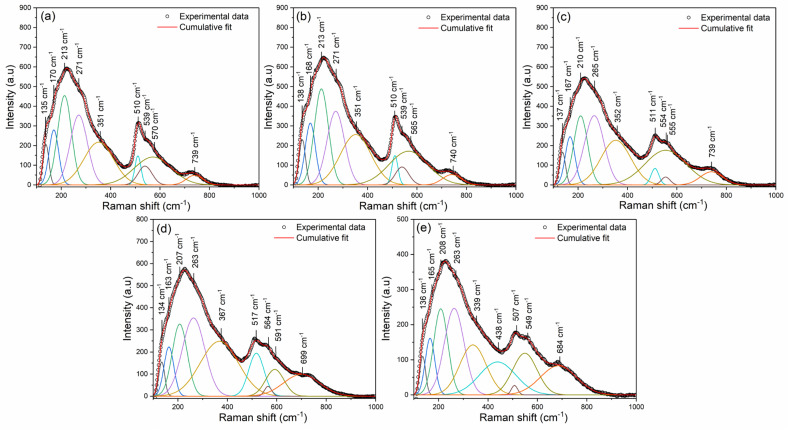
Raman spectra of (Ba_0.7_Sr_0.3_)_1 − *x*_Nd*_x_*Ti_1 − *y*_Mn*_y_*O_3_ ceramics for (**a**) *x* = 0 and *y* = 0, (**b**) *x* = 0.005 and *y* = 0, (**c**) *x* = 0.005 and *y* = 0.0025, (**d**) *x* = 0.005 and *y* = 0.005, and (**e**) *x* = 0.005 and *y* = 0.01.

Raman spectra of BSNTM ceramics in the range of 100–1000 cm^−1^ are shown in Figure 3. The Raman bands of all samples indicate the tetragonal phase of the perovskite structure in BST ceramics, which is similar to that in BST-based reports [39,47]. The spectral parameters of the Raman modes, such as the Raman shift of the central position of each peak and corresponding full width at half maxima (FWHM), are calculated by fitting the Gaussian function. A total of nine Raman active modes were observed. The modes that appeared around 135 and 168 cm^−1^ are associated with the vibration of A-site cations (A-O); 213, 271, and 351 cm^−1^ are related to the vibrations of B-O; 510, 539, and 565 cm^−1^ are related to the vibrations of BO_6_; and 740 cm^−1^ corresponds to the *A*_1_ + E (LO) overlapping modes [39]. The mode at 135 cm^−1^ is slightly shifted to a higher wavenumber of 138 cm^−1^ with Nd substitution for *x* = 0.005 and *y* = 0. This is caused by an *A*-site disorder, which is attributed to the incorporation of Nd^3+^ at Ba^2+^ and Sr^2+^ ions. The modes around 271 and 539 cm^−1^ shifted towards a lower wavenumber with an increasing Mn concentration from *x* = 0.005 and *y* = 0.0025 to 0.01. This is due to an increase in the *B*-site disorder in the BSNTM related to the creation of lattice tensile stress due to lattice expansion [48]. These results are well supported via XRD, dielectric, and ferroelectric properties.

### 3.2. Microstructural Properties

**Figure 4:**
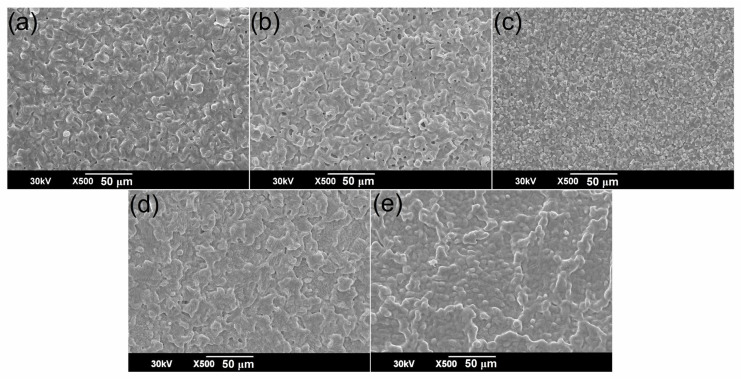
FESEM images of (Ba_0.7_Sr_0.3_)_1 − *x*_Nd*_x_*Ti_1 − *y*_Mn*_y_*O_3_ ceramics for (**a**) *x* = 0 and *y* = 0, (**b**) *x* = 0.005 and *y* = 0, (**c**) *x* = 0.005 and *y* = 0.0025, (**d**) *x* = 0.005 and *y* = 0.005, and (**e**) *x* = 0.005 and *y* = 0.01.

FESEM images of the BSNTM ceramics are shown in Figure 4. The *x* = 0.005 and *y* = 0.01 sample shows a uniform microstructure and has a more compact grain size distribution compared to that of pure BST and other samples of BSNTM (*x =* 0.005 and *y* < 0.01). The density of BSNTM ceramics was estimated using the Archimedes principle to confirm a dense and uniform microstructure. The estimated relative density was found to be in the range of 91% to 98% of the theocratical density, thus verifying that all the samples had a highly dense and uniform microstructure. The average grain size of the BSNTM (*x* = 0 and *y* = 0) was found to be 3.59 µm and was reduced to 1.99 µm with the substitution of Nd and Mn co-dopants in BSNTM for *x* = 0.005 and *y* = 0.01. The reduction in grain size with a uniform microstructure was due to the formation of oxygen vacancies caused by Mn^2+^ occupying Ti^4+^. Soo and Qiaoli et al. [47,49] reported that Sm and Yb, and Nd and Mn co-doped BT ceramics with donor/donor–acceptor defect complexes via charge compensation/oxygen vacancy exhibited a uniform and small-grained microstructure. Smaller grains with uniform and dense microstructures can resist higher voltages, which results in a high BDS and enhanced energy storage properties [50,51].

**Figure 5:**
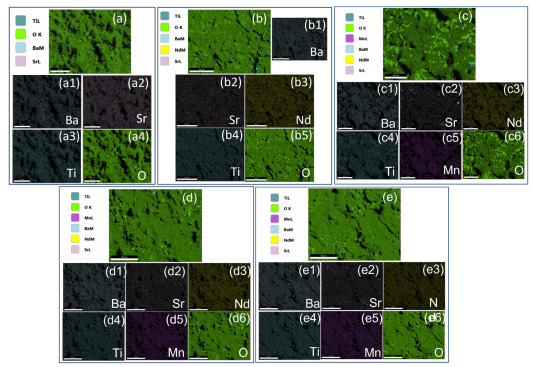
Typical microstructure (**a**–**e**) and elemental mapping (a1–a4, b1–b5, c1–c6, d1–d6, and e1–e6) of (Ba_0.7_Sr_0.3_)_1 − *x*_Nd*_x_*Ti_1 − *y*_Mn*_y_*O_3_ ceramics for (**a**) *x* = 0 and *y* = 0, (**b**) *x* = 0.005 and *y* = 0, (**c**) *x* = 0.005 and *y* = 0.0025, (**d**) *x* = 0.005 and *y* = 0.005, and (**e**) *x* = 0.005 and *y* = 0.01.

**Figure 6:**
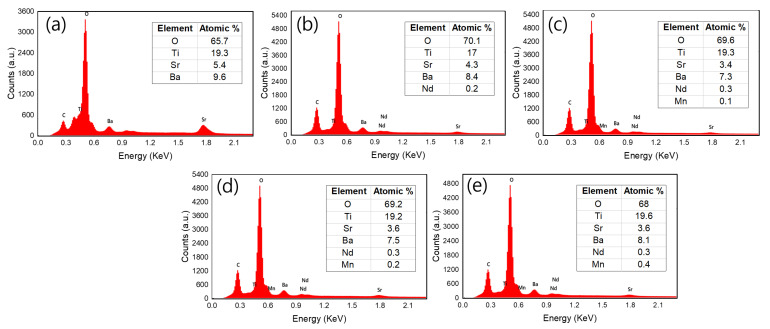
EDX spectra of (Ba_0.7_Sr_0.3_)_1 − *x*_Nd*_x_*Ti_1 − *y*_Mn*_y_*O_3_ ceramics for (**a**) *x* = 0 and *y* = 0, (**b**) *x* = 0.005 and *y* = 0, (**c**) *x* = 0.005 and *y* = 0.0025, (**d**) *x* = 0.005 and *y* = 0.005, and (**e**) *x* = 0.005 and *y* = 0.01.

SEM–energy-dispersive X-ray spectroscopy (EDX) elemental mapping was analyzed to show the incorporation and distribution of doping elements. Figure 5 shows the typical microstructure (a–e) and elemental mapping (a1–a4, b1–b5, c1–c6, d1–d6, and e1–e6) of (Ba_0.7_Sr_0.3_)_1-*x*_Nd*_x_*Ti_1-*y*_Mn*_y_*O_3_ ceramics for all samples. The elemental mapping results show the existence of elements Ba, Sr, Nd, Ti, Mn, and O, suggesting that Nd and Mn elements enter grains and are distributed homogeneously. The measured experimental volume fraction of BSNTM composition is approximately equal to the theoretical volume fractions, confirming the stoichiometry of BSNTM (Figure 6).

### 3.3. Dielectric Properties

**Figure 7:**
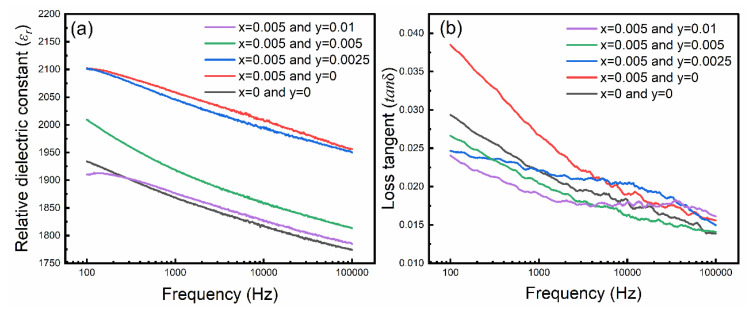
(**a**) Relative dielectric constant and (**b**) loss tangent as a function of the frequency of (Ba_0.7_Sr_0.3_)_1 − *x*_Nd*_x_*Ti_1 − *y*_Mn*_y_*O_3_ ceramics.

The relative dielectric constant (*ε_r_*) and the loss tangent (*tanδ*) of BSNTM ceramic capacitors measured as a function frequency at RT from 100 Hz to 100 kHz are shown in Figure 7. Pure BST (*x* = 0 and *y* = 0) exhibited a *ε_r_* of 1868 and *tanδ* of 0.0218 at 1 kHz, which increased to 2058 and 0.0266 with Nd substitution for *x* = 0.005 and *y* = 0. Further, these values gradually decreased to 1876 and 0.0191 with Mn substitution into BSNTM for *x* = 0.005 and *y* = 0.01. The Nd and Mn co-dopants in the BST matrix favored the formation of donor–acceptor complexes. Nd^3+^ ions in a BST system can compensate for the formation of oxygen vacancies, leading to the enhancement of the dielectric properties (*ε_r_* and *tanδ*) of BSNT ceramics. On the other hand, Mn^2+^ ions in the BSNT system facilitate the formation of oxygen vacancies, prevent a decrease in Ti^4+^, and yield a low *ε_r_* and *tanδ* [47].

### 3.4. P-E Loops and Energy Storage Performance

**Figure 8:**
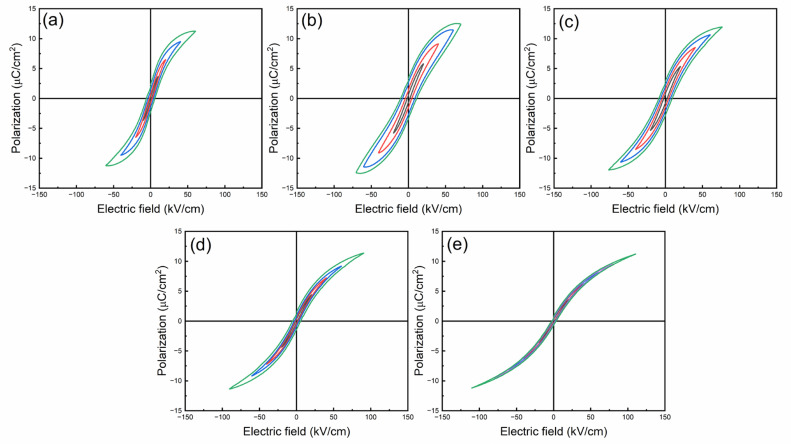
RT bipolar *P-E* loops of (Ba_0.7_Sr_0.3_)_1 − *x*_Nd*_x_*Ti_1 − *y*_Mn*_y_*O_3_ ceramics measured under different electric fields at 10 Hz for (**a**) *x* = 0 and *y* = 0, (**b**) *x* = 0.005 and *y* = 0, (**c**) *x* = 0.005 and *y* = 0.0025, (**d**) *x* = 0.005 and *y* = 0.005, and (**e**) *x* = 0.005 and *y* = 0.01.

*P-E* loops of BSNTM ceramics measured at RT under different electric fields at a frequency of 10 Hz are shown in Figure 8. The ferroelectric BSNTM (*x* = 0.005 and *y* = 0) ceramics displayed a large maximum polarization, *P_max_*, of 12.5 µC/cm^2^, a small remnant polarization, *P_r_*, of 3.35 µC/cm^2^ (i.e., Δ*P* = 9.15 µC/cm^2^), and a high coercive field, *E_c_*, of 11.2 kV/cm. The *P_max_*, *P_r_*, and *E_c_* values gradually reduced, Δ*P* and *E_BD_* values increased from 9.15 to 10.39 µC/cm^2^, and 70.6 to 110.6 kV/cm from *x* = 0.005 and *y* = 0 to *x* = 0.005 and *y* = 0.01, as shown in Table 2. The BSNTM sample for *x* = 0.005 and *y* = 0.01 (Figure 8e) exhibits a slim saturated *P-E* loop, and the improved *E_BD_* is attributed to the decrease in grain size and defect dipoles generated with the incorporation of Mn at the Ti site of the BST host lattice; this can be understood via Kroger–Vink notation as follows [46,52]:

(4) $${Mn}_{2}O_{3}\overset{\left(2TiO_{2}\right)}{\to }{2Mn}_{Ti}^{\prime }+{3O}_{o}+V_{o}^{∙∙}$$

(5) $$MnO\overset{\left(TiO_{2}\right)}{\to }\to {Mn}_{Ti}^{\prime }+O_{o}+V_{o}^{∙∙}$$

This shows that the oxygen vacancies are generated by Mn^3+^ and Mn^2+^ replacing Ti^4+^ at the B-site. In Equations (4) and (5), 2Ti^4+^ needs four lattice oxygen O_o_ to maintain charge neutrality, whereas 2Mn^3+^ requires 3O_o_. When 2Mn^3+^ substitutes at 2Ti^4+^, 1O_o_ is released as ½ O_2_, generating oxygen vacancies, $V_{o}^{∙∙}$, with two positive charges. Thus, Mn^2+^ replaces Ti^4+^ (Equation (5)) [46].

**Figure 9:**
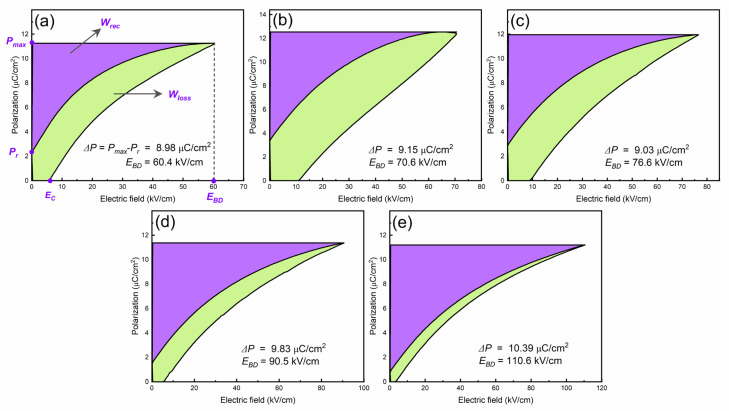
RT unipolar *P-E* loops of (Ba_0.7_Sr_0.3_)_1 − *x*_Nd*_x_*Ti_1 − *y*_Mn*_y_*O_3_ ceramics measured at 10 Hz for (**a**) *x* = 0 and *y* = 0, (**b**) *x* = 0.005 and *y* = 0, (**c**) *x* = 0.005 and *y* = 0.0025, (**d**) *x* = 0.005 and *y* = 0.005, and (**e**) *x* = 0.005 and *y* = 0.01.

The *W_rec_* and *η* values of BSNTM ceramic capacitors were derived from *P-E* loops using Equations (2) and (3), as shown in Table 2. The *W_rec_* and *η* values gradually increased with the increasing Mn concentration, and the sample with *x* = 0.005 and *y* = 0.01 exhibited a high energy density of 0.41 J/cm^3^ at an *E_BD_* of 110.6 kV/cm, and a high energy efficiency of 84.6%, as shown in Figure 9e. The enhancement in the energy storage properties is realized using defect dipole engineering via the co-doping of Nd and Mn in BST (mostly governed by Mn). ${(Mn}_{Ti}^{”}-V_{O}^{∙∙}{)}^{∙}$ and ${(Mn}_{Ti}^{”}-V_{O}^{∙∙})$ defect dipoles between acceptor ions and oxygen vacancies can capture electrons and improve BDS. In addition, the defect dipoles act as a driving force for depolarization, making it possible to design domain formation energy and domain wall energy, which provides a high difference between *P_max_* and *P_r_* (Δ*P* = 10.39 µC/cm^2^) [46]. Moreover, complex defect dipoles with optimum oxygen vacancies can provide not only a high Δ*P*, but also reduce grain size, which together improve breakdown strength with Mn and lead to a high energy storage density and high energy efficiency in BSNTM ceramics. It is well known that Δ*P* and *E_BD_* are key factors for energy storage performance, i.e., higher Δ*P* and *E_BD_* values account for huge energy storage density and efficiency [53].

**Figure 10:**
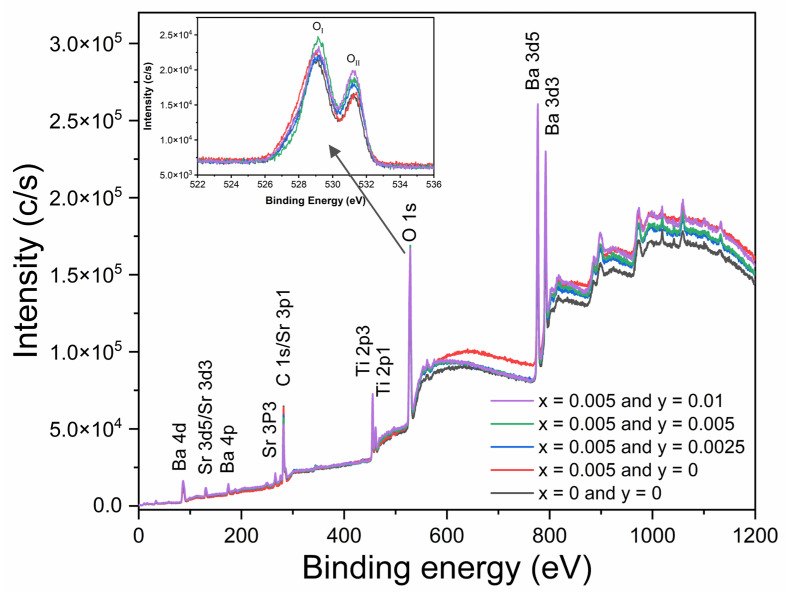
XPS spectra of (Ba_0.7_Sr_0.3_)_1 − *x*_Nd*_x_*Ti_1 − *y*_Mn*_y_*O_3_ ceramics. Inset figure represents high-resolution XPS spectra of O 1s.

X-ray photoelectron spectroscopy (XPS) measurement was carried out to show the chemical states of Nd- and Mn-doped BST ceramics. Figure 10 shows the XPS spectra of BSNTM ceramics. Twelve peaks in terms of binding energy were detected, which correspond to those of Ba, Sr, Ti, O and C elements. Among these, C may arise from carbon pollution in the air and instruments. However, no peaks were detected for Nd and Mn elements due to the very low doping concentration (*x* = 0.005 and *y* = 0.0025–0.01). The inset of Figure 10 shows the high-resolution XPS spectra of O 1s, which was divided into two distinct peaks: the first peak (O_I_) found at 529.08 eV indicates an oxygen lattice, and the second peak (O_II_) at 531.16 eV is related to oxygen vacancies [54]. The relative intensity of the BSNTM ceramics increases with Mn doping, and the *x* = 0.005 and *y* = 0.01 sample shows much a stronger relative intensity than that of other samples, which clearly indicates that the sample processes high oxygen vacancies (inset Figure 10) [55,56].

## 4. Conclusions

In summary, we demonstrated a defect dipole engineering method to improve the breakdown strength and energy storage properties by co-doping Nd and Mn in BST ceramics, which are fabricated via a traditional solid-stated reaction method. The XRD and Raman spectra of all samples revealed a tetragonal crystal structure. FESEM images of BSNTM ceramics exhibit a uniform and dense microstructure, whereas the average grain size decreases with an increasing Mn concentration. In addition, the dielectric properties decreased with Mn due to the formation of oxygen vacancies, which were confirmed via XPS analysis. Moreover, the complex defect dipoles with smaller grain sizes and lower dielectric losses provided a high difference between *P_max_* and *P_r_*, and improved the breakdown strength with Mn, leading to high energy density and efficiency in the BSNTM ceramics. These features suggest that defect dipole engineering is an effective approach to enhance energy storage performance for pulsed-power capacitor applications.

## Figures and Tables

**Table 1:** Rietveld refined XRD parameters of (Ba_0.7_Sr_0.3_)_1 − *x*_Nd*_x_*Ti_1 − *y*_Mn*_y_*O_3_ ceramics (Note: Tetra: tetragonal and Tri: triclinic).

Composition	Lattice Parameters (Å)		V (Å^3^)	Phase Fraction (%)	χ^2^
	Tetragonal (P4 mm)	Triclinic (P1)			
*x* = 0 and *y* = 0	a = b = 3.97377 ± 0.00007 c = 3.98820 ± 0.00023 α = β = γ = 90°	a = 9.74235 ± 0.00045 b = 10.06442 ± 0.00025 c = 10.13491 ± 0.00020 α = 91.43°, β = 91.31° and γ = 96.38°	V_Tetra_ = 62.977 ± 0.004 V_Tri_ = 986.928 ± 0.055	Tetra = 94.41 Tri = 5.59	1.95
*x* = 0.005 and *y* = 0	a = b = 3.97989 ± 0.00022 c = 3.94835 ± 0.00064 α = β = γ = 90°	a = 9.71450 ± 0.00034 b = 10.045162 ± 0.00022 c = 10.12407 ± 0.00022 α = 91.41°, β = 91.22° and γ = 96.44°	V_Tetra_ = 62.510 ± 0.011 V_Tri_ = 981.114 ± 0.046	Tetra = 92.92 Tri = 7.08	2.67
*x* = 0.005 and *y* = 0.0025	a = b = 3.97339 ± 0.00017 c = 3.97732 ± 0.00025 α = β = γ = 90°	a = 9.72015 ± 0.00035 b = 10.06498 ± 0.00026 c = 10.13280 ± 0.00017 α = 91.46°, β = 91.28° and γ = 96.36°	V_Tetra_ = 62.793± 0.005 V_Tri_ = 984.566± 0.047	Tetra = 87.73 Tri = 12.27	2.05
*x* = 0.005 and *y* = 0.005	A = b = 3.97485 ± 0.00016 C = 3.96880 ± 0.00032 A = β = γ = 90°	a =9.71171±0.00039 b = 10.06801±0.00023 c = 10.13123±0.00022 α = 91.46°, β = 91.26° and γ = 96.38°	V_Tetra_ = 62.705± 0.006 V_Tri_ = 983.839± 0.050	Tetra = 94.03 Tri = 5.97	2.02
*x* = 0.005 and *y* = 0.01	a = b=3.97102 ± 0.00026 c = 3.97123 ± 0.00023 α = β = γ =90°	a = 9.70581 ± 0.00027 b = 10.06558 ± 0.00018 c = 10.12693 ± 0.00017 α = 91.45°, β = 91.25° and γ = 96.38°	V_Tetra_ = 62.622 ± 0.007 V_Tri_ = 982.577 ± 0.037	Tetra = 95.16 Tri = 4.84	1.77

**Table 2:** Ferroelectric and energy storage parameters of BSNTM ceramics.

Composition	*P_r_* (µC/cm^2^)	*P_max_* (µC/cm^2^)	*ΔP = P_max_−P_r_*	*E_c_* (kV/cm)	*E_BD_* (kV/cm)	*W_rec_* (J/cm^3^)	*η* (%)
*x* = 0 and *y* = 0	2.22	11.2	8.98	5.94	60.4	0.15	48.5
*x* = 0.005 and *y* = 0	3.35	12.5	9.15	11.2	70.6	0.19	38.8
*x* = 0.005 and *y* = 0.0025	2.87	11.9	9.03	9.34	76.6	0.22	48.9
*x* = 0.005 and *y* = 0.005	1.57	11.4	9.83	5.45	90.5	0.3	69.4
*x* = 0.005 and *y* = 0.01	0.81	11.2	10.39	3.09	110.6	0.41	84.6

## Data Availability

Not applicable.
